# Comprehensive spectral libraries for various rabbit eye tissue proteomes

**DOI:** 10.1038/s41597-022-01241-5

**Published:** 2022-03-29

**Authors:** Guoting Qin, Pengzhi Zhang, Mingxia Sun, Wenjiang Fu, Chengzhi Cai

**Affiliations:** 1 College of Optometry, University of Houston, Houston, TX 77204 USA; 2 Mass Spectrometry Laboratory, Department of Chemistry, University of Houston, Houston, TX 77204 USA; 3 Department of Physics, University of Houston, Houston, TX 77204 USA; 4 Department of Mathematics, University of Houston, Houston, TX 77204 USA

**Keywords:** Physiology, Zoology

## Abstract

Rabbits have been widely used for studying ocular physiology and pathology due to their relatively large eye size and similar structures with human eyes. Various rabbit ocular disease models, such as dry eye, age-related macular degeneration, and glaucoma, have been established. Despite the growing application of proteomics in vision research using rabbit ocular models, there is no spectral assay library for rabbit eye proteome publicly available. Here, we generated spectral assay libraries for rabbit eye compartments, including conjunctiva, cornea, iris, retina, sclera, vitreous humor, and tears using fractionated samples and ion mobility separation enabling deep proteome coverage. The rabbit eye spectral assay library includes 9,830 protein groups and 113,593 peptides. We present the data as a freely available community resource for proteomic studies in the vision field. Instrument data and spectral libraries are available via ProteomeXchange with identifier PXD031194.

## Abstract (summary)

**Table:** 

Measurement(s)	database type spectral library
Technology Type(s)	ion mobility spectrometry-mass spectrometry
Sample Characteristic - Organism	Oryctolagus cuniculus
Sample Characteristic - Environment	eye
Sample Characteristic - Location	United States of America

## Background & Summary

A wide variety of ocular diseases lead to severe visual impairments and ultimately to blindness. Vision impairments and loss have substantial economic implications. Conservative assessments suggest that annual global productivity loss from vision impairment for 2020 is approximately US$410.7 billion purchasing power parity^1^. Rabbit ocular models have been used for advancing the understanding of human ocular diseases and the development of therapeutics^2–4^. Due to their relatively large eye size and anatomical and histological similarity with human eyes^5^, rabbit models have been developed for dry eye^6,7^, age-related macular degeneration^8,9^, and glaucoma^10–12^, perioperative corneal abrasion^13^, retinal detachment^14^, lens regeneration^15^, wound healing^16^, and other ocular diseases. Rabbit ocular models are also used to study the effect of surgical intervention in ophthalmology, such as lensectomy^17^, subretinal prostheses^18^, and cataract surgery^19^. A comprehensive review of rabbit ocular models was reported by Zernii, *et al*.^20^.

Mass spectrometry-based proteomics has led to the possibility of characterizing and quantifying the protein profile of complex biological systems; therefore, it has been widely used in biomarker discovery, disease diagnosis, and treatment monitoring^21–25^. In bottom-up proteomics, a spectral library is often used to identify and accurately quantify tens to hundreds of thousands of peptides, especially for data-independent acquisition (DIA)-mass spectrometry^26^. Consistent quantification and deep proteome profiling of complex proteomic samples require a comprehensive spectral library.

Shotgun proteomics has been used in studying ocular physiology and pathology using rabbit models^3,6,14–17,19,27–29^. However, there is no spectral assay library for rabbit eye proteome publicly available. The eye is a highly compartmentalized organ with unique features, such as transparency of the cornea and light reception by the retina. Therefore, individual spectral libraries were generated for rabbit conjunctiva, cornea, iris, retina, sclera, tears, and vitreous humor. From 108 fractionated various ocular compartments using a timsTOF Pro mass spectrometer, we generated a comprehensive spectral library that can be used for the identification of 113,593 peptides mapping to 9,830 rabbit ocular proteins. The quality of all libraries was assessed using the spectral library tool DIALib-QC^30^. All spectral libraries are transferrable to other instruments collecting DIA data and other software identifying and quantitating peptides and proteins. All instrument data and spectral libraries have been deposited to the ProteomeXchange^31^ Consortium (http://proteomecentral.proteomexchange.org) via the PRIDE^32^ partner repository with the dataset identifier PXD031194^33^.

## Methods

### Materials and reagents

LC-MS grade water, acetonitrile (ACN), formic acid (FA), and sequencing grade trypsin were purchased from Thermo Fisher Scientific (Pittsburgh, PA, USA). iRT peptides were purchased from Biognosys AG (Switzerland). All other chemicals were purchased from Millipore Sigma (St. Louis, MO) and used without further purification unless noted otherwise.

### Rabbit eye tissue collection

All animal handling was in accordance with the ARVO Statement for the Use of Animals in Ophthalmic and Vision Research and approved by the Institutional Animal Care and Use Committee at the University of Houston. Eye tissues from four eyes of two New Zealand White rabbits (2–3 kg, female, 9–18 months) were collected, and pooled for proteomic profiling. The rabbits were healthy in general, and had no known prior eye complications such as infection, inflammation, etc., and no prior eye surgeries. Briefly, 40 μL of sterile saline was instilled onto each eye, and tears were collected without anesthesia. Within 1 h of post euthanasia, eye tissues - cornea, conjunctiva, iris, retina, sclera, and vitreous humor (Table 1) - were extracted. Care was taken not to contaminate each tissue sample with the adjacent structures. All tissue samples were placed in sterile 1.5 mL tubes and stored at −80 °C until further processing.

**Table 1:** Sample overview.

Sample type	Fractions	Library name
Conjunctiva	16	RabbitConjunctiva
Cornea	16	RabbitCornea
Iris	16	RabbitIris
Retina	16	RabbitRetina
Sclera	16	RabbitSclera
Tears	12	RabbitTears
Vitreous Humor	16	RabbitVitreousHumor
Total	108	RabbitEye

Sample types, numbers of fractions after high pH reverse phase fractionation, and names of the generated spectral assay libraries were included.

### Peptide sample preparation

Equal volumes of 100 mM ammonium bicarbonate were added to the tear samples and the samples were heated at 95 °C for 5 min. The reduction and alkylation reactions were carried out by adding 5 mM dithiothreitol at 37 °C for 1 h and 10 mM iodoacetamide for 30 min in the dark, respectively. Protein concentration was determined using Bradford reagent, and 50 μg of proteins were used for tryptic digestion. Trypsin (1.25 μg, 1/40, w/w) was then added and incubated at 37 °C overnight. The reaction was stopped by adding trifluoroacetic acid (TFA). The digested peptides were cleaned up using C18 Ziptips and vacuum dried using a CentriVap (Labconco Corporation, Kansas City, MO).

Vitreous humor samples were centrifuged at 15000 × g at 4 °C for 15 min. Then, the supernatant was transferred to a clean tube, added to an equal volume of 100 mM ammonium bicarbonate, and heated at 95 °C for 5 min. The reduction, alkylation, digestion, and clean-up procedures were carried out as above.

The entire part of all other tissue samples (cornea, conjunctiva, iris, retina, and sclera) was used to ensure deep proteome coverage for each tissue^34^. Each tissue sample was minced into fine pieces in a biosafety level II cabinet and collected into clean 1.5 mL tubes. To each tube was added 100 μL of 100 mM ammonium bicarbonate (30% ACN, 8 M urea, and 20 mM dithiothreitol)^35^. After incubation at 37 °C for 30 min under mild shaking, the samples were centrifuged at 15,000 × g for 15 min. The supernatant was transferred to a clean tube, and 100 mM iodoacetamide was added. After incubation in the dark for 30 min at room temperature, 1 mL of 100 mM ammonium bicarbonate was added to each sample to reduce the urea concentration. The digestion and clean-up procedures were carried out as above. Other methods such as homogenization, sonication, and cryogenic grinding may be used for sample processing before protein extraction to improve protein coverage.

### High pH reverse-phase fractionation

Dried peptide samples were resuspended in 10 mM ammonium formate (AF) pH 10. Stage-tips containing 6 C18 membranes were used to fractionate peptides. The stage-tips were pre-treated with isopropanol and 60% ACN in 10 mM AF pH 10, and finally re-equilibrated using 10 mM AF pH 10. Samples were then loaded onto the stage-tips, washed twice using 10 mM AF pH 10, and eluted into 12 or 16 fractions using an escalating concentration of ACN (2–60%) in 10 mM AF pH 10. Fractions were dried before reconstitution in 2% ACN with 0.1% FA for analysis and spiked with iRT peptides according to the manufacturer’s instruction^36^.

### nanoLC-MS/MS

The liquid chromatography-mass spectrometry procedure was published elsewhere^37^. Specifically, a NanoElute LC system coupled to a timsTOF Pro (Bruker Daltonics, Germany) via a CaptiveSpray source was used. Samples were loaded onto an in-house packed column (75 μm x 25 cm, 1.9 μm ReproSil-Pur C18 particle (Dr. Maisch GmbH, Germany), column temperature 40 °C) with buffer A (0.1% FA in water) and buffer B (0.1% FA in ACN) as mobile phases. The 120-min gradient was 60 min from 2% B to 17% B, 90 min to 25% B, 100 min to 37% B, 110 min to 80% B, and maintained for another 10 min. The parallel accumulation-serial fragmentation (PASEF) mode with 10 PASEF scans per cycle was used. The electrospray voltage was 1.4 kV, and the ion transfer tube temperature was 180 °C. Full MS scans were acquired over the mass-to-charge (m/z) range of 150–1700. The target intensity value was 2.0 × 10^5^ with a threshold of 2500. A fixed cycle time was set to 1.2 s, and a dynamic exclusion duration was 0.4 min with ± 0.015 amu tolerance. Only peaks with charge state ≥ 2 were selected for fragmentation.

### Data processing

Software Spectronaut^38^ v15 (Biosynosis, Switzerland) was used with default settings to generate spectral libraries. The UniProt SwissProt and TrEMBL combined database^39^ (*Oryctolagus cuniculus* (Taxon ID 9986), downloaded on 01/10/2022, 43,526 entries containing both reviewed (894) and unreviewed (42632) entries without isoforms) was used to build the spectral library. Cysteine carbamidomethylation was used as a fixed modification, and methionine oxidation and acetylation as variable modifications. The false discovery rate (FDR) was controlled at < 1% at peptide spectrum match, peptide, and protein levels. Spectral assay libraries were generated for each rabbit eye tissue type (Table 1). In total, 108 data-dependent acquisition raw mass spectrometry data were used to generate one combined spectral assay library.

### Spectral assay library quality control using DIALib-QC

All generated spectral assay libraries were evaluated using DIALib-QC^30^ (http://www.swathatlas.org/DIALibQC.php), a freely available software tool to evaluate a library’s characteristics, completeness, and correctness across 62 parameters of compliance. The DIALib-QC assessment reports for all spectral libraries have been deposited to the ProteomeXchange Consortium^31^ (http://proteomecentral.proteomexchange.org) via the PRIDE^32^ partner repository with the dataset identifier PXD031194^33^.

## Data Records

The raw mass spectrometry data (.d), generated spectral assay library files (.xls,.kit), and DIALib-QC assessment reports for all spectral libraries have been deposited to the ProteomeXchange Consortium via PRIDE^32^ with the dataset identifier PXD031194^33^. The data will be shared under the terms of the Creative Commons Attribution (CC BY) license as per PRIDE’s standard terms. The raw mass spectrometry data files were labelled as “Rabbit(name of the eye tissue)_(other information such as fraction number, etc.).d.zip”. The spectral libraries were labelled as “Rabbit(name of the eye tissue)Library.kit” and “Rabbit(name of the eye tissue)Library.xls”. The.kit files can be imported into Spectronaut software and used for DIA-MS data analysis. The spectral libraries in generic format (.xls) can be used by third-party software such as Skyline. All DIALib-QC assessment reports for all spectral libraries were compressed into one zip file, within which quality assessment reports for each individual spectral library were included.

## Technical Validation

**Fig. 1:**
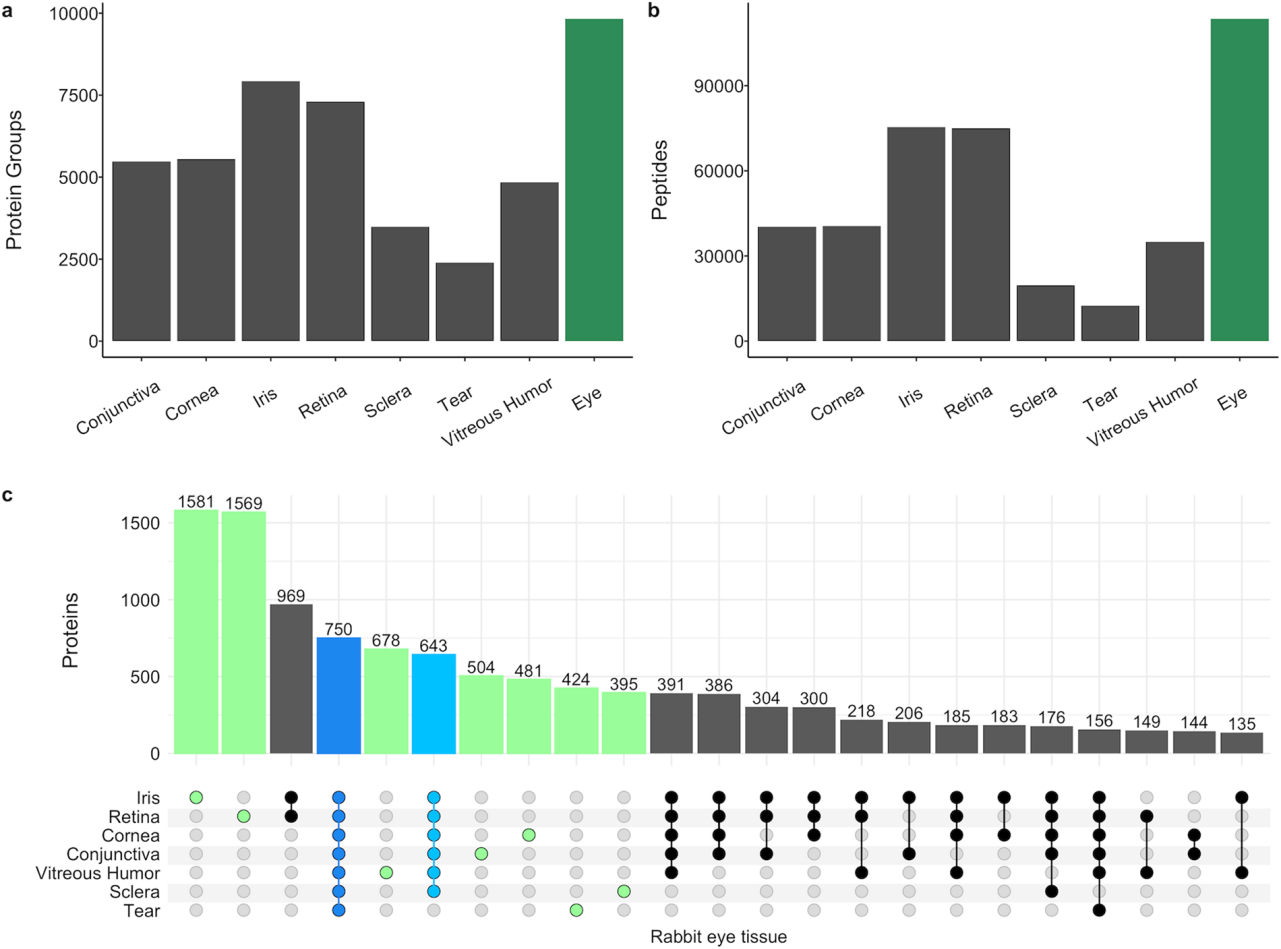
Spectral assay library characteristics. The number of proteins (**a**) and peptides (**b**) in each rabbit ocular tissue spectral assay library (dark gray) and the rabbit eye library (dark green). (**c**) Bar graph showing the overlaps and uniquely identified proteins in each category as denoted by the dot matrix. Each dot in solid colors except light gray represents proteins present in the corresponding ocular tissue shown at the left of the matrix. The number of proteins identified in all 7 ocular tissues is highlighted in blue, and the number of proteins identified in 6 ocular tissues (except tear) is highlighted in light blue. The number of proteins uniquely identified in each ocular tissue is highlighted in green.

High-quality assay libraries are required for accurate identification and precise quantification of peptides and proteins. To generate a spectral library for each rabbit ocular compartment, the DDA-MS datasets described above (see Methods and Data records, Table 1) were analyzed using the Biognosys’ proprietary search engine Pulsar. False identifications are controlled by FDR estimation at three levels: peptide-spectrum match (PSM), peptide, and protein group level. To generate the spectral library for the entire eye, the search results (PSMs) from each compartment analysis before applying any FDR filter were combined, and a library-wide control of FDR was applied at the PSM, peptide, and protein group level. In all libraries (Table 1), the FDR was controlled at 1% at all three levels. There were 12,468 peptides representing 2,398 protein groups in the rabbit tear spectral library, which is the smallest library in all compartments (Fig. 1a,b). There were 75,384 peptides representing 7,927 protein groups in the rabbit iris spectral library, the largest library in all compartments (Fig. 1a,b). Overall, the rabbit eye spectral library included 2,214,258 transitions identifying 149,074 peptide precursors representing 113,593 stripped peptides and 9,830 protein groups (dark green bar in Fig. 1a,b). Among the spectral libraries for each rabbit eye compartment, 750 protein groups were common in all eye compartments, and 643 protein groups were common in all eye compartments except for tears (Fig. 1c), which accounted for 14% of the proteins identified in the rabbit eye. In addition, there were 1,581, 1,569, 678, 504, 481, 424, and 395 protein groups that were uniquely identified in rabbit iris, retina, vitreous humor, conjunctiva, cornea, tear, and sclera, respectively (Fig. 1c). These high numbers of proteins unique to each eye tissue demonstrated the differences between compartments.

**Fig. 2:**
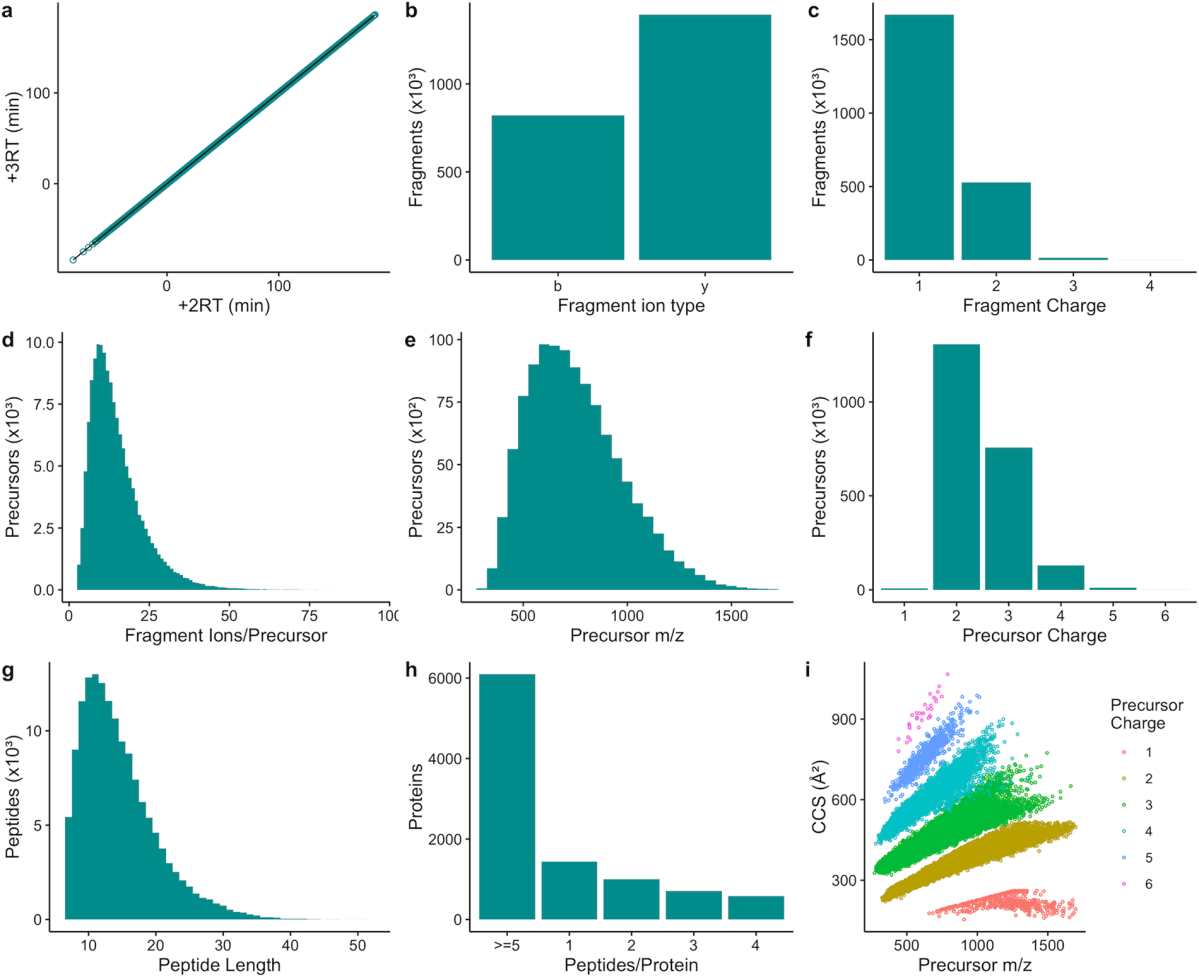
Quality assessment of the rabbit eye spectral assay library. (**a**) Retention time (RT) fit of +2 and +3 charge states of the sample peptides in the spectral library. (**b**) Number of observed b- and y- ion fragments in the spectral library. (**c**) Distribution of fragment ions by observed fragment charge states in the spectral library. (**d**) Distribution of precursors by the number of fragment ions per precursor. (**e**) Distribution of precursors by precursor m/z values across the acquired mass range in the assay library. (**f**) Distribution of precursors by precursor charge states. (**g**) Distribution of the observed peptides by peptide length in the assay library. (**h**) Distribution of proteins by the number of peptides per protein in the spectral library. (**i**) Distribution of precursor CCS values across the acquired mass range in the spectral library color-coded by precursor charge state.

To ensure correct identification of peptides and proteins during DIA-MS data analysis, DIALib-QC was used to evaluate the quality and characteristics of the library. As shown in Fig. 2a, high RT similarity between + 2 and +3 charge states of the same peptide as indicated by an RT correlation r^2^ value of 1 demonstrates the high quality of chromatography and retention time normalization based on reference peptides in the library. Fig. 2b showed a higher number of y than b fragment ions (63% vs. 37%), which is expected with collision-induced dissociation (CID) fragmentation. Moreover, more than 99% of fragment ions are with +1 or +2 charge states (Fig. 2c). Peptides with more than 6 fragment ions per precursor constitute more than 90% of the library, ensuring an adequate number of ions to estimate peptide quantities (Fig. 2d). Precursor m/z values in the library range from 150 to 1,700 m/z with 60% of the precursors in the range between 400 and 1200 (Fig. 2e). Precursor charge states range from + 1 to +6, among which 99% are of charge states between +2 and +4 (Fig. 2f). The length of the peptides ranges from 7 to 46 amino acids, with > 98% of the total of less than 30 amino acids in length (Fig. 2g). Proteins with more than 5 peptides per protein constitute 62% of the proteins in the library, and the proteins with 2 or more peptides per protein reach 85% of the total (Fig. 2h). The high number of peptides per protein ensures confident identification of such proteins in the DIA-MS data analysis. The ion mobility values in the library were converted into ion-neutral collisional cross section (CCS) values using the Mason-Schamp equation^40^. Within each charge state, CCS values are correlated with m/z values (Fig. 2i). The ion mobility values in the library improve identification confidence of peptides, thus proteins in the data analysis.

## Usage Notes

### Library search of MS/MS data

Spectrum annotation via library search is both faster and more sensitive than database search algorithms^41^. Due to a lack of data, library search has not been practical except for the most common species (e.g., human and bacteria). As the assay libraries presented here contain data for several major tissues in the rabbit eye, peptide and protein identification via spectral library matching in vision research using rabbit models becomes an attractive alternative to database searching. Moreover, the use of the CCS values has the potential to increase the confidence in the identification of peptides.

### Compatibility with commonly used software for peptide and protein analysis

In this study, we provide spectral assay libraries in Spectronaut’s native format (.kit) and generic (.xls) format. The spectral libraries in generic format can be used with commonly used software such as Skyline for peptide and protein analysis.

## Data Availability

Software used in the generation of this project is third-party software as described in the Data Records section, i.e., Spectronaut and DIALib-QC.

## References

[1] Burton MJ (2021). The Lancet Global Health Commission on Global Eye Health: vision beyond 2020. Lancet Glob. Health.

[2] Park HYL, Kim JH, Lee KM, Park CK (2012). Effect of prostaglandin analogues on tear proteomics and expression of cytokines and matrix metalloproteinases in the conjunctiva and cornea. Exp. Eye Res..

[3] Qiu XD, Gong L, Sun XH, Guo JC, Chodara AM (2011). Efficacy of Acupuncture and Identification of Tear Protein Expression Changes Using iTRAQ Quantitative Proteomics in Rabbits. Curr. Eye Res..

[4] del Amo EM, Urtti A (2015). Rabbit as an animal model for intravitreal pharmacokinetics: Clinical predictability and quality of the published data. Exp. Eye Res..

[5] Van Cruchten S (2017). Pre- and Postnatal Development of the Eye: A Species Comparison. Birth Defects Res..

[6] Zhou L (2013). Proteomic analysis revealed the altered tear protein profile in a rabbit model of Sjogren’s syndrome-associated dry eye. Proteomics.

[7] Xiong C (2008). A rabbit dry eye model induced by topical medication of a preservative benzalkonium chloride. Invest. Ophthalmol. Vis. Sci..

[8] Pennesi ME, Neuringer M, Courtney RJ (2012). Animal models of age related macular degeneration. Mol. Asp. Med..

[9] Li Y (2018). A novel model of persistent retinal neovascularization for the development of sustained anti-VEGF therapies. Exp. Eye Res..

[10] Ozgonul C, Mumcuoglu T (2015). Rabbit Model of Trabeculectomy for Glaucoma Surgery Researches. J. Clin. Anal. Med..

[11] Evangelho, K., Mastronardi, C. A. & de-la-Torre, A. Experimental Models of Glaucoma: A Powerful Translational Tool for the Future Development of New Therapies for Glaucoma in Humans-A Review of the Literature. *Med. Lith*. **55** (2019). 10.3390/medicina55060280 PMC6630440 31212881

[12] Hou B (2020). Study of minimally invasive radiofrequency ablation of the ciliary body for the treatment of glaucoma in rabbits. Mol. Med. Rep..

[13] Zernii EY (2017). Mechanisms of Perioperative Corneal Abrasions: Alterations in the Tear Film Proteome. Biochem. Mosc.-Suppl. B-Biomed. Chem..

[14] Mandal, N. *et al*. Proteomic Analysis of the Vitreous following Experimental Retinal Detachment in Rabbits. *J. Ophthalmol*. **2015** (2015). 10.1155/2015/583040 PMC4667062 26664739

[15] Liu XL (2008). Proteomic analysis of regenerated rabbit lenses reveal crystallin expression characteristic of adult rabbits. Mol. Vis..

[16] Zhou L (2007). Proteomic analysis of rabbit tear fluid: Defensin levels after an experimental corneal wound are correlated to wound closure. Proteomics.

[17] Young JB (2020). Quantitative proteomic analysis of aqueous humor after rabbit lensectomy reveals differences in coagulation and immunomodulatory proteins. Mol. Omics.

[18] Xiao, Y. *et al*. Acute Rabbit Eye Model for Testing Subretinal Prostheses. *Transl. Vis. Sci. Technol*. **8** (2019). 10.1167/tvst.8.5.20 PMC6779096 31602345

[19] Stastna, M., Behrens, A., McDonnell, P. J. & Van Eyk, J. E. Analysis of protein composition of rabbit aqueous humor following two different cataract surgery incision procedures using 2-DE and LC-MS/MS. *Proteome Sci*. **9** (2011). 10.1186/1477-5956-9-8 PMC3045281 21306621

[20] Zernii EY (2016). Rabbit Models of Ocular Diseases: New Relevance for Classical Approaches. CNS Neurol. Disord.-Drug Targets.

[21] Aebersold R, Mann M (2003). Mass spectrometry-based proteomics. Nature.

[22] Anjo, S. I., Santa, C. & Manadas, B. SWATH-MS as a tool for biomarker discovery: From basic research to clinical applications. *Proteomics* **17** (2017). 10.1002/pmic.201600278 28127880

[23] Qian WJ, Jacobs JM, Liu T, Camp DG, Smith RD (2006). Advances and challenges in liquid chromatography-mass spectrometry-based proteomics profiling for clinical applications. Mol. Cell. Proteomics.

[24] Meyer JG, Schilling B (2017). Clinical applications of quantitative proteomics using targeted and untargeted data-independent acquisition techniques. Expert Rev. Proteomics.

[25] Sajic T, Liu YS, Aebersold R (2015). Using data-independent, high-resolution mass spectrometry in protein biomarker research: Perspectives and clinical applications. Proteom. Clin. Appl..

[26] Collins, B. C. *et al*. Multi-laboratory assessment of reproducibility, qualitative and quantitative performance of SWATH-mass spectrometry. *Nat. Commun*. **8** (2017). 10.1038/s41467-017-00249-5 PMC5566333 28827567

[27] Miller I, Rogel-Gaillard C, Spina D, Fontanesi L, de Almeida AM (2014). The Rabbit as an Experimental and Production Animal: From Genomics to Proteomics. Curr. Protein Pept. Sci..

[28] Liu, Y., Bouhenni, R. A., Dufresne, C. P., Semba, R. D. & Edward, D. P. Differential Expression of Vitreous Proteins in Young and Mature New Zealand White Rabbits. *PLoS One* **11** (2016). 10.1371/journal.pone.0153560 PMC4835093 27089221

[29] Stastna M (2007). Proteomics of the aqueous humor in healthy New Zealand rabbits. Proteomics.

[30] Midha, M. K. *et al*. DIALib-QC an assessment tool for spectral libraries in data-independent acquisition proteomics. *Nat. Commun*. **11** (2020). 10.1038/s41467-020-18901-y PMC7567827 33067471

[31] Deutsch EW (2020). The ProteomeXchange consortium in 2020: enabling ‘big data’ approaches in proteomics. Nucleic Acids Res..

[32] Perez-Riverol Y (2019). The PRIDE database and related tools and resources in 2019: improving support for quantification data. Nucleic Acids Res..

[33] Qin GT, Zhang PZ, Sun MX, Fu WJ, Cai CZ (2022). PRIDE Archive.

[34] Liu Y (2017). Anatomical differences of the protein profile in the rabbit sclera during growth. Exp. Eye Res..

[35] Dapic I, Uwugiaren N, Jansen PJ, Corthals GL (2017). Fast and Simple Protocols for Mass Spectrometry-Based Proteomics of Small Fresh Frozen Uterine Tissue Sections. Anal. Chem..

[36] Escher C (2012). Using iRT, a normalized retention time for more targeted measurement of peptides. Proteomics.

[37] Zhu ZL (2020). Ortho-Substituted alpha-Phenyl Mannoside Derivatives Promoted Early-Stage Adhesion and Biofilm Formation of E. coli 83972. ACS Appl. Mater. Interfaces.

[38] Bruderer R (2015). Extending the Limits of Quantitative Proteome Profiling with Data-Independent Acquisition and Application to Acetaminophen-Treated Three-Dimensional Liver Microtissues. Mol. Cell. Proteomics.

[39] Bateman A (2019). UniProt: a worldwide hub of protein knowledge. Nucleic Acids Res..

[40] Mason, E. A. & McDaniel, E. W. *Transport Properties of Ions in Gases*. (John Wiley & Sons, Inc., 1988).

[41] Zhang X, Li YZ, Shao WG, Lam H (2011). Understanding the improved sensitivity of spectral library searching over sequence database searching in proteomics data analysis. Proteomics.

